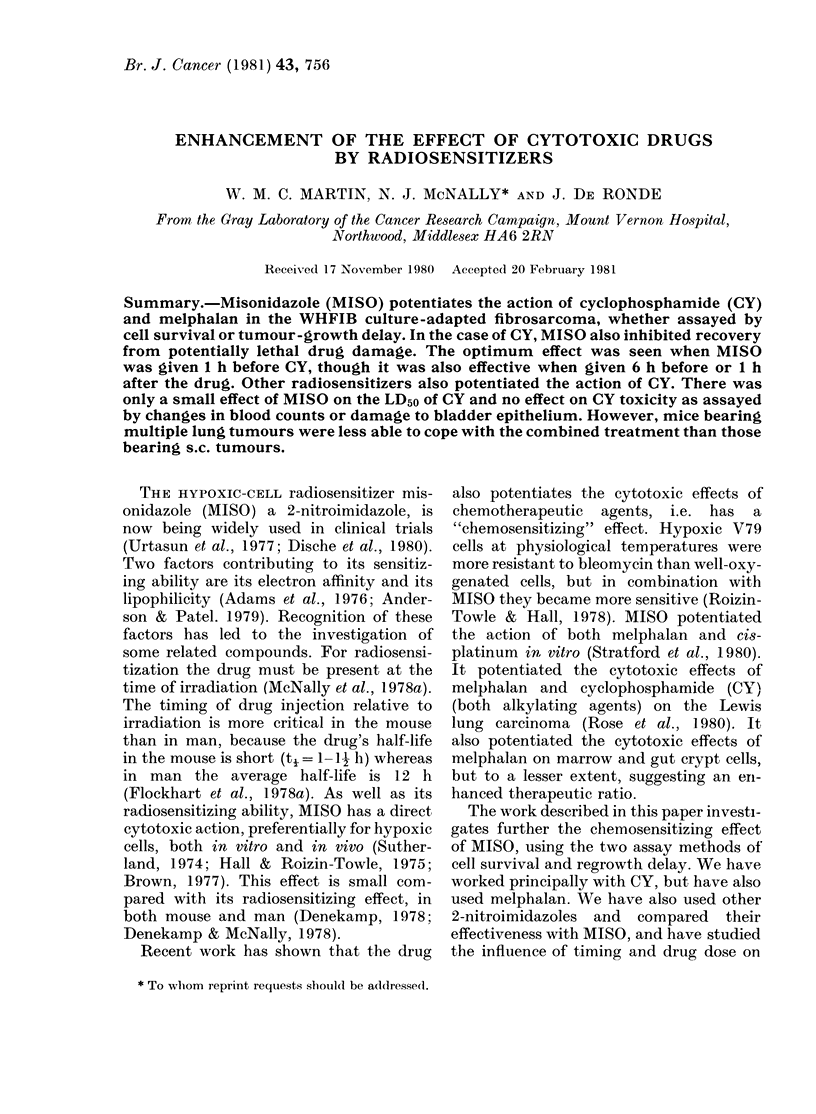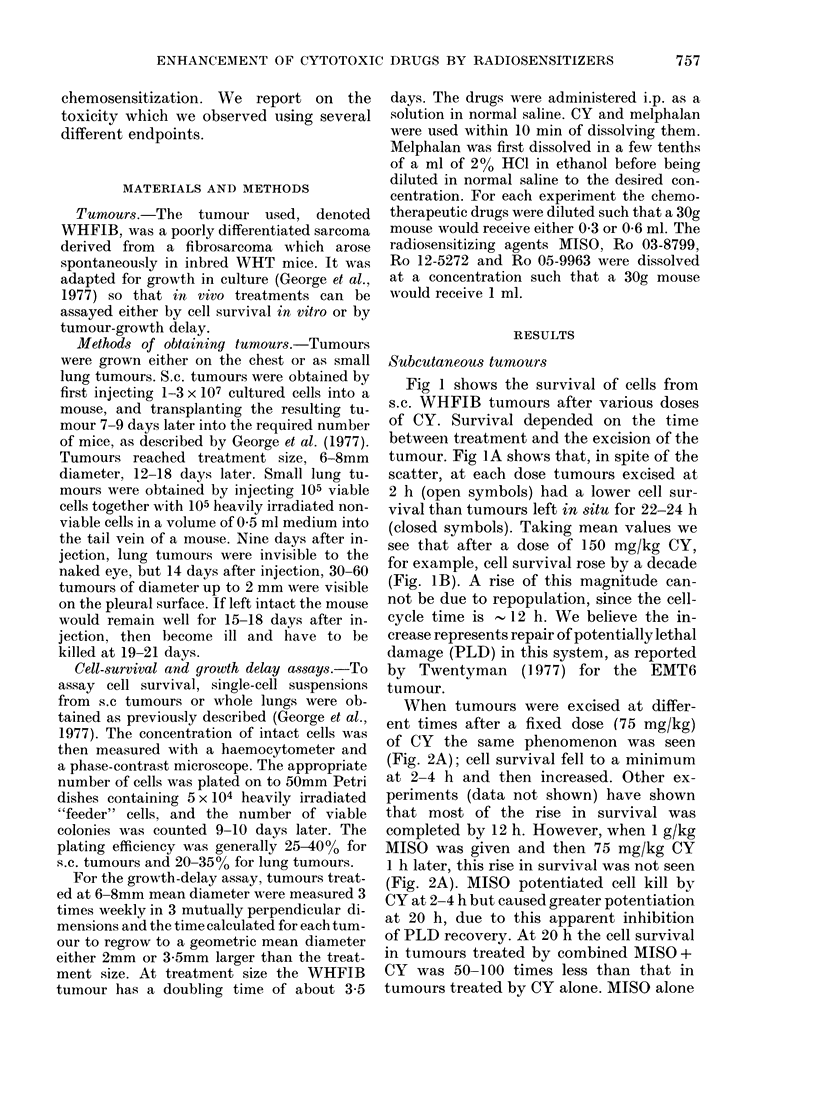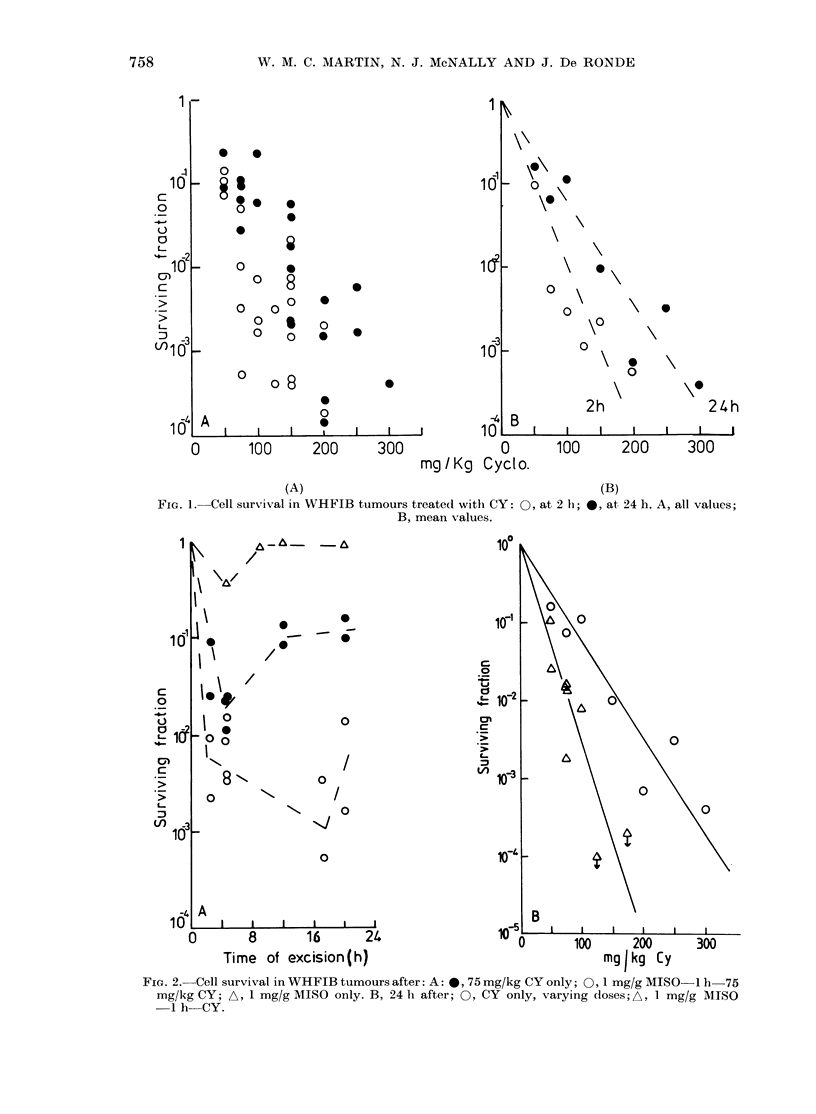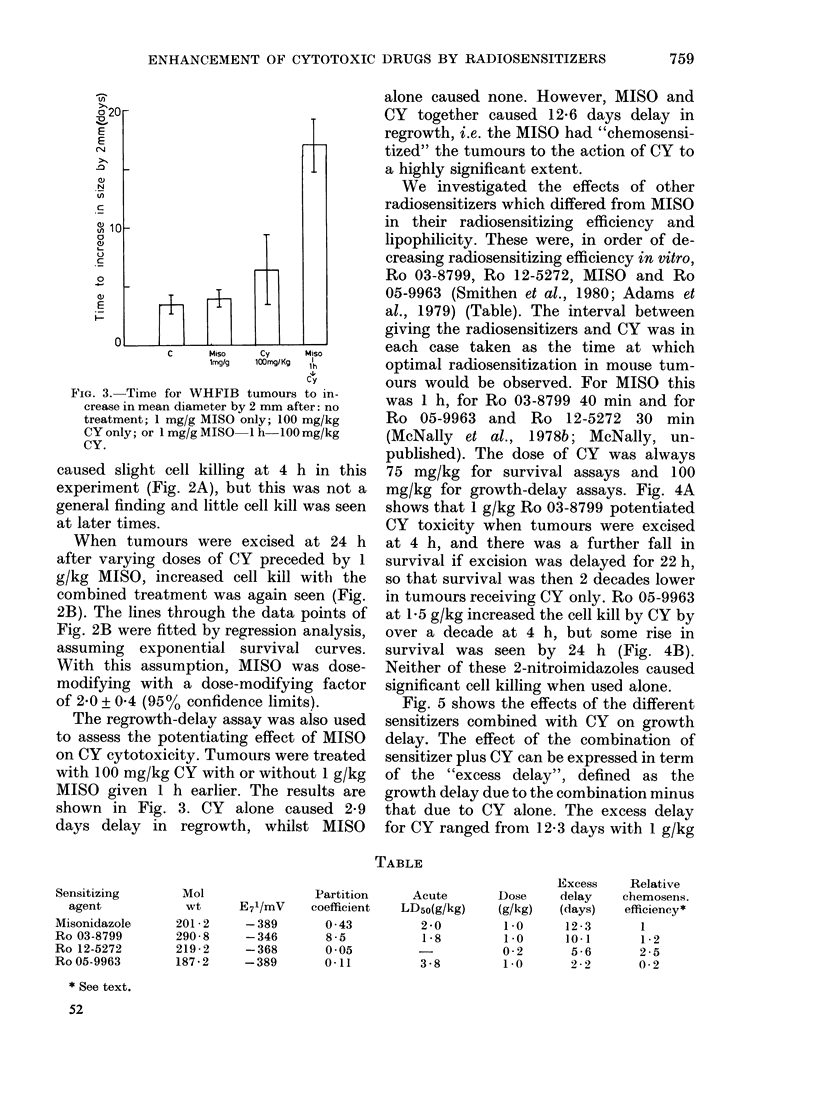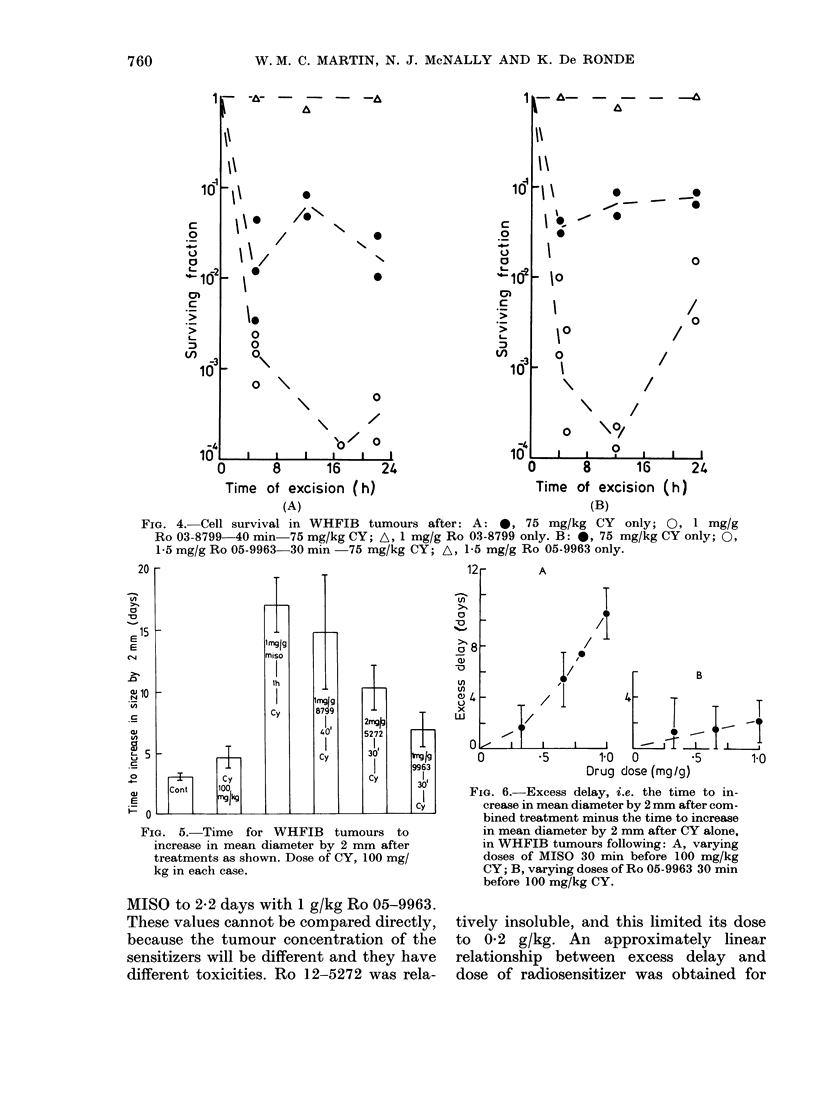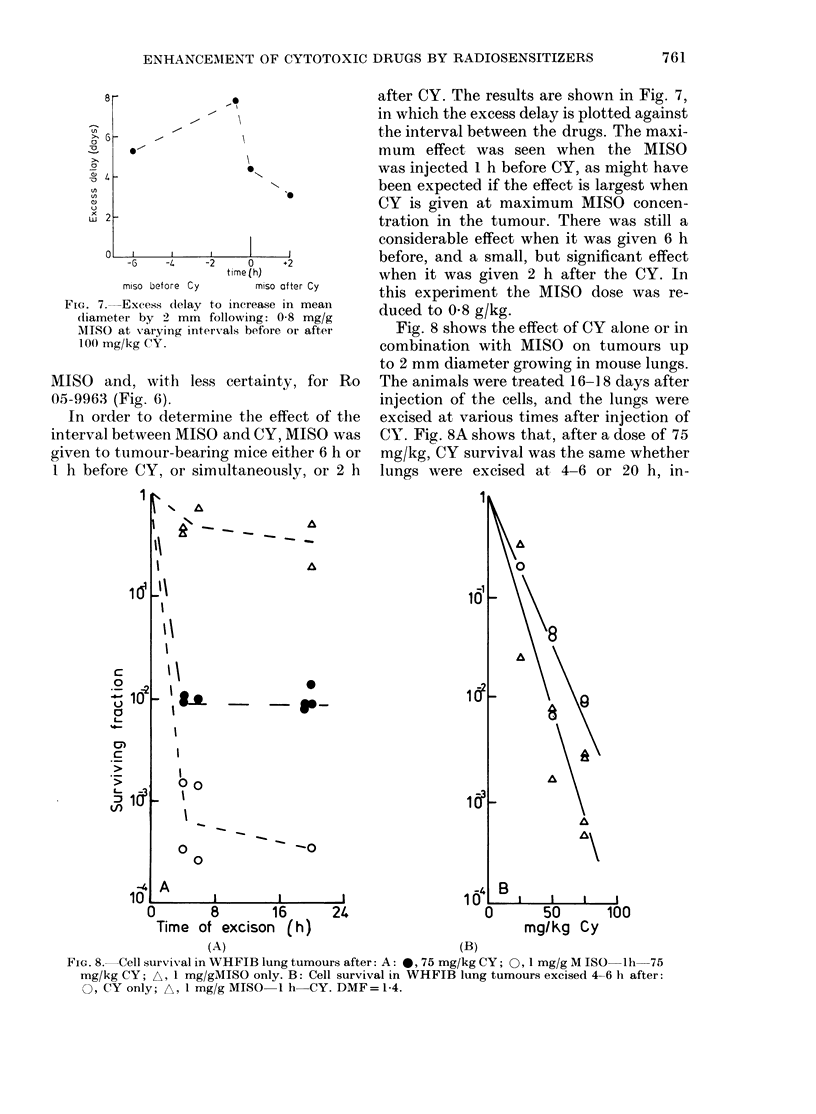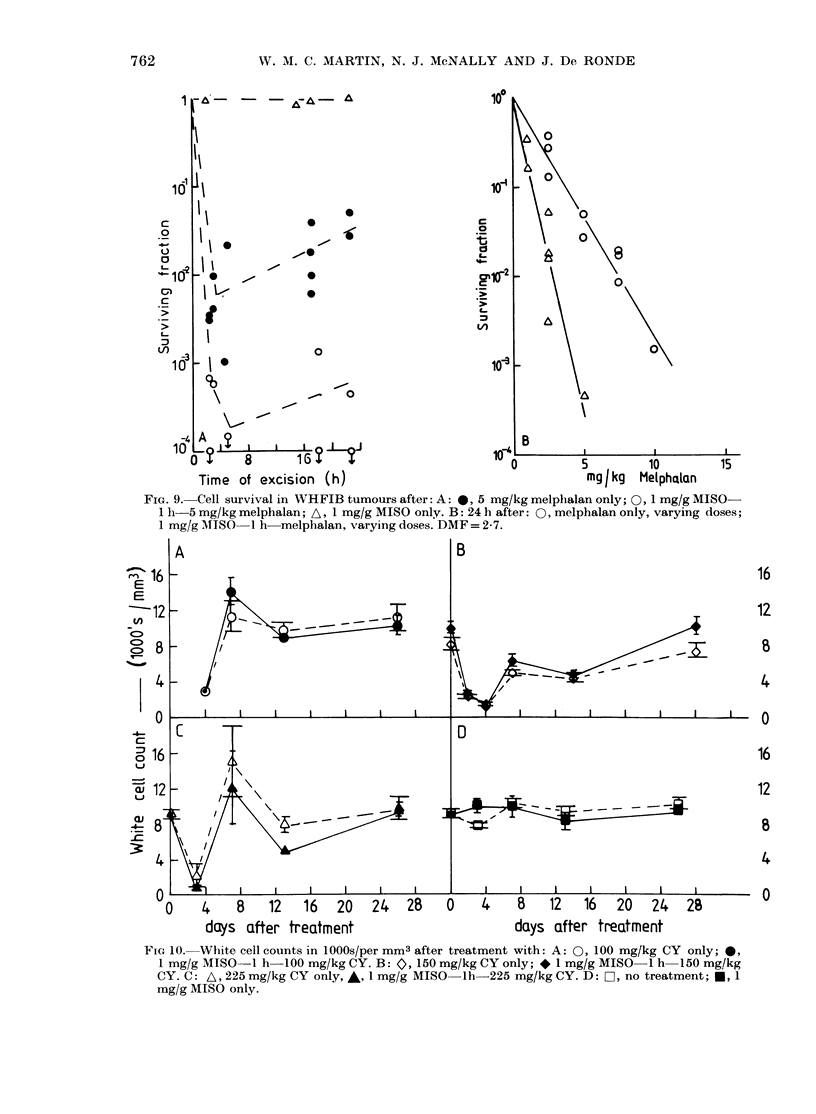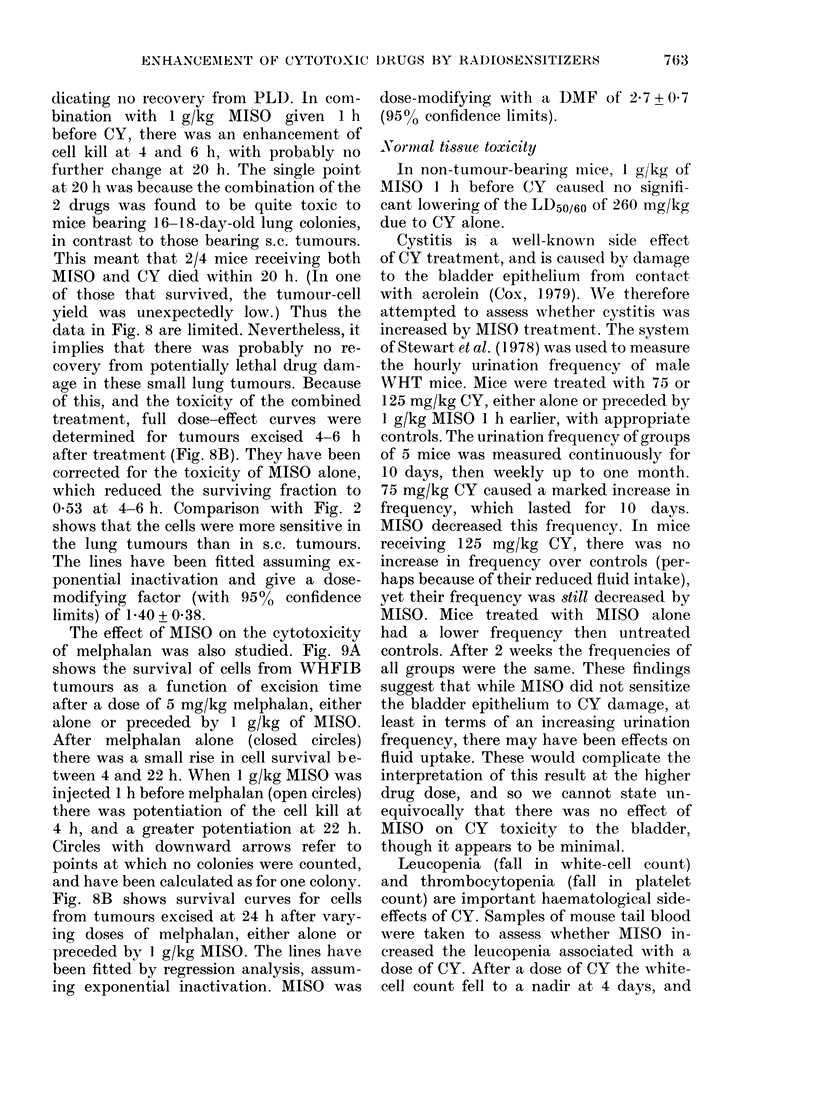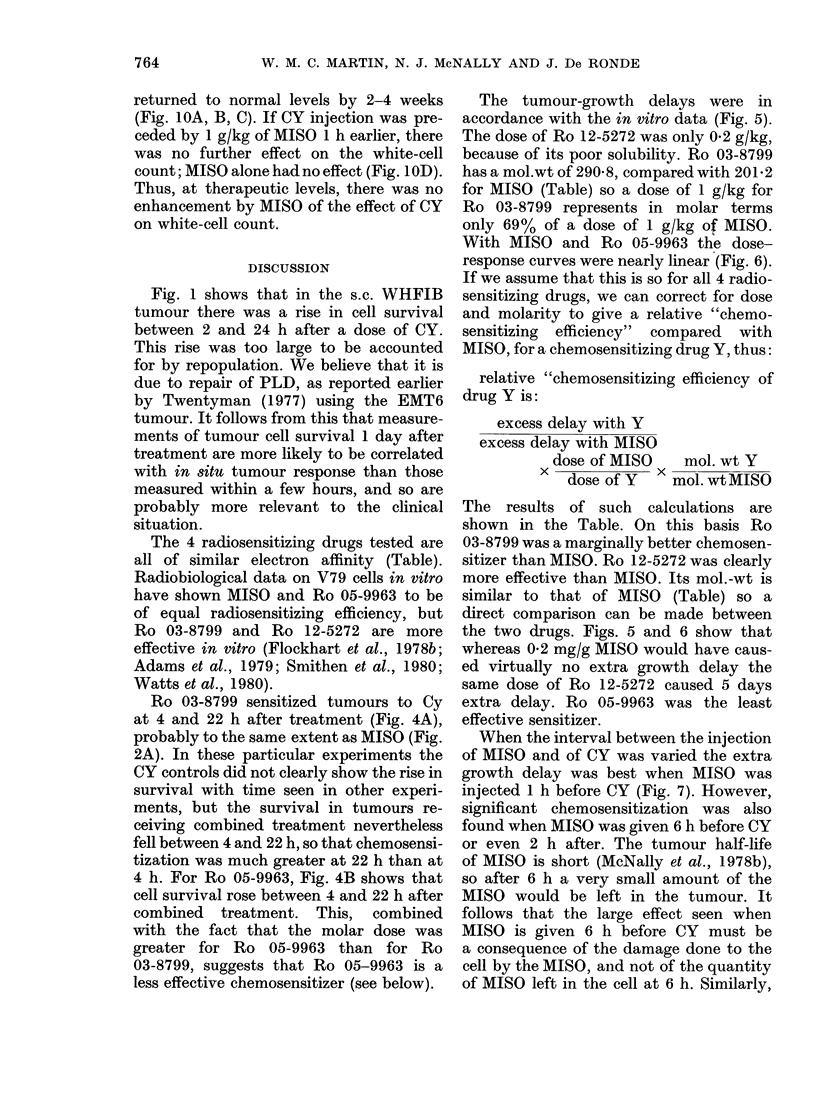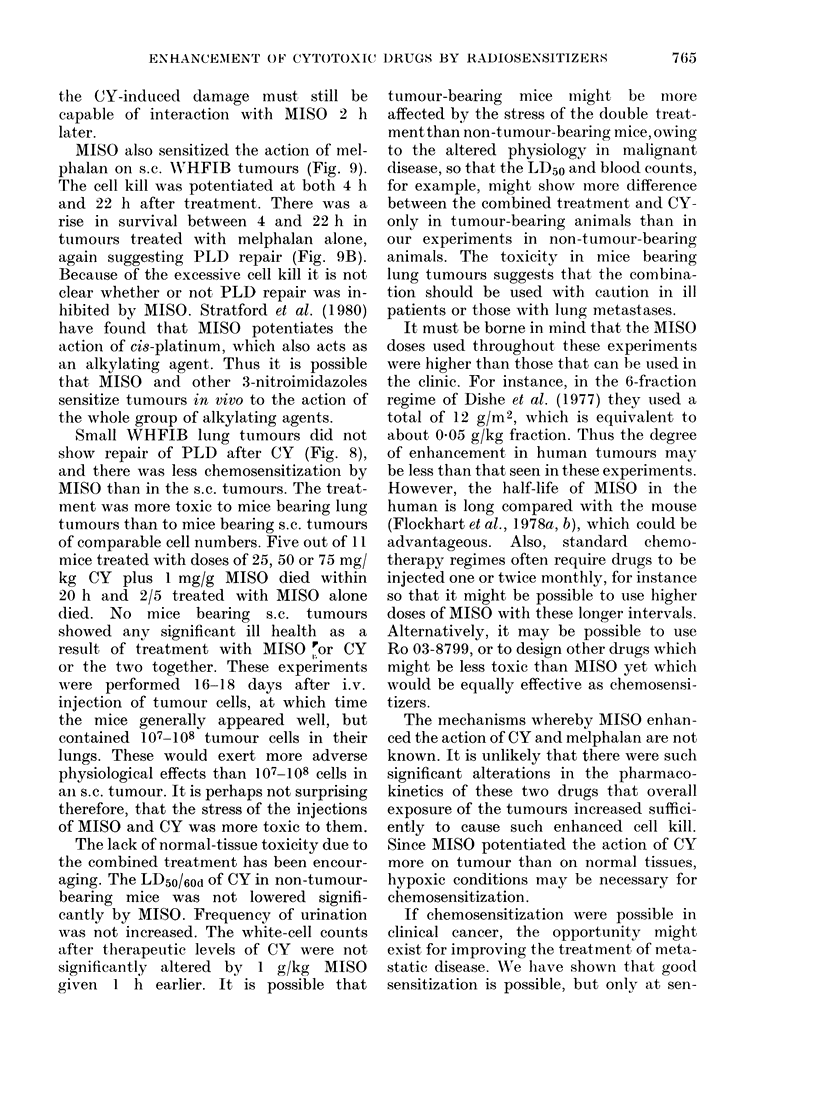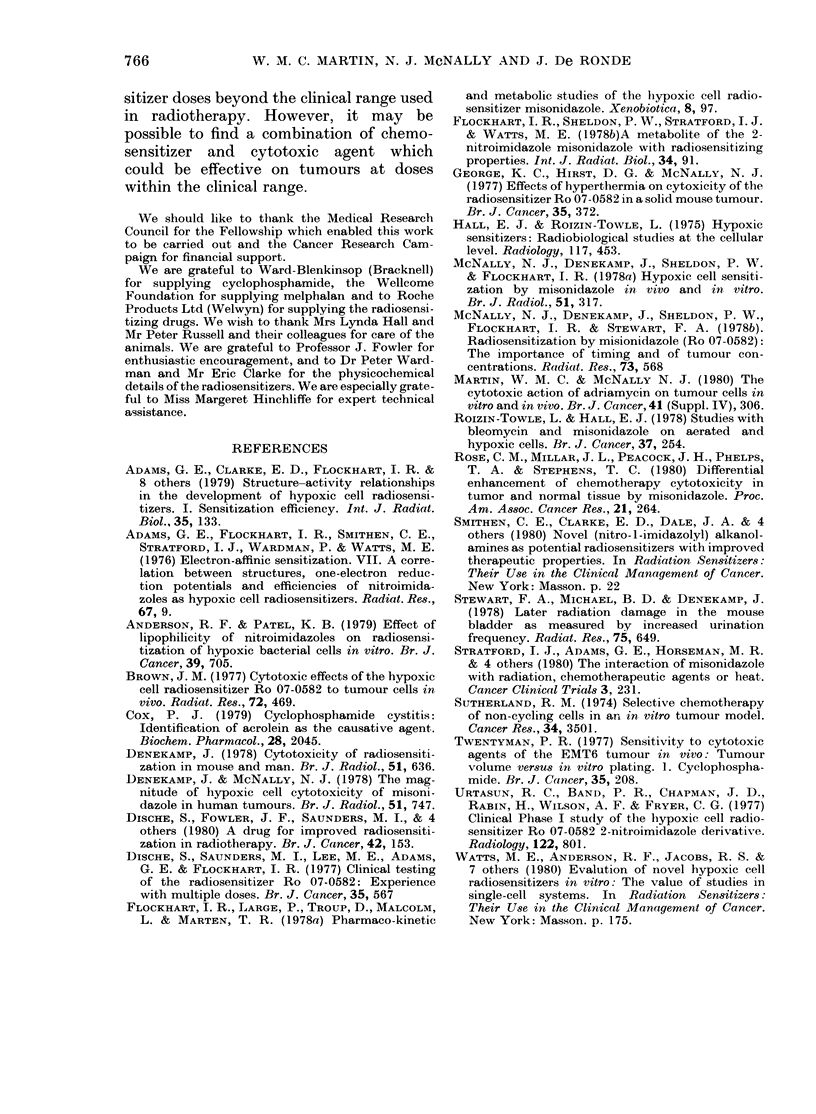# Enhancement of the effect of cytotoxic drugs by radiosensitizers.

**DOI:** 10.1038/bjc.1981.113

**Published:** 1981-06

**Authors:** W. M. Martin, N. J. McNally, J. De Ronde

## Abstract

Misonidazole (MISO) potentiates the action of cyclophosphamide (CY) and melphalan in the WHFIB culture-adapted fibrosarcoma, whether assayed by cell survival or tumour-growth delay. In the case of CY, MISO also inhibited recovery from potentially lethal drug damage. The optimum effect was seen when MISO was given 1 h before CY, though it was also effective when given 6 h before or 1 h after the drug. Other radiosensitizers also potentiated the action of CY. There was only a small effect of MISO on the LD50 of CY and no effect on CY toxicity as assayed by changes in blood counts or damage to bladder epithelium. However, mice bearing multiple lung tumours were less able to cope with the combined treatment than those bearing s.c. tumours.


*Br. J. Cancer* (1981) **43**, 756

### ENHANCEMENT OF THE EFFECT OF CYTOTOXIC DRUGS BY RADIOSENSITIZERS

W. M. C. MARTIN, N. J. McNALLY* AND J. DE RONDE

*From the Gray Laboratory of the Cancer Research Campaign, Mount Vernon Hospital, Northwood, Middlesex HA6 2RN*

Received 17 November 1980 Accepted 20 February 1981

**Summary.—Misonidazole (MISO) potentiates the action of cyclophosphamide (CY) and melphalan in the WHFIB culture-adapted fibrosarcoma, whether assayed by cell survival or tumour-growth delay. In the case of CY, MISO also inhibited recovery from potentially lethal drug damage. The optimum effect was seen when MISO was given 1 h before CY, though it was also effective when given 6 h before or 1 h after the drug. Other radiosensitizers also potentiated the action of CY. There was only a small effect of MISO on the $LD_{50}$ of CY and no effect on CY toxicity as assayed by changes in blood counts or damage to bladder epithelium. However, mice bearing multiple lung tumours were less able to cope with the combined treatment than those bearing s.c. tumours.**

THE HYPOXIC-CELL radiosensitizer misonidazole (MISO) a 2-nitroimidazole, is now being widely used in clinical trials (Urtasun *et al.*, 1977; Dische *et al.*, 1980). Two factors contributing to its sensitizing ability are its electron affinity and its lipophilicity (Adams *et al.*, 1976; Anderson & Patel. 1979). Recognition of these factors has led to the investigation of some related compounds. For radiosensitization the drug must be present at the time of irradiation (McNally *et al.*, 1978*a*). The timing of drug injection relative to irradiation is more critical in the mouse than in man, because the drug's half-life in the mouse is short ($t_{\frac{1}{2}} = 1$–$1\frac{1}{2}$ h) whereas in man the average half-life is 12 h (Flockhart *et al.*, 1978*a*). As well as its radiosensitizing ability, MISO has a direct cytotoxic action, preferentially for hypoxic cells, both *in vitro* and *in vivo* (Sutherland, 1974; Hall & Roizin-Towle, 1975; Brown, 1977). This effect is small compared with its radiosensitizing effect, in both mouse and man (Denekamp, 1978; Denekamp & McNally, 1978).

Recent work has shown that the drug also potentiates the cytotoxic effects of chemotherapeutic agents, i.e. has a "chemosensitizing" effect. Hypoxic V79 cells at physiological temperatures were more resistant to bleomycin than well-oxygenated cells, but in combination with MISO they became more sensitive (Roizin-Towle & Hall, 1978). MISO potentiated the action of both melphalan and *cis*-platinum *in vitro* (Stratford *et al.*, 1980). It potentiated the cytotoxic effects of melphalan and cyclophosphamide (CY) (both alkylating agents) on the Lewis lung carcinoma (Rose *et al.*, 1980). It also potentiated the cytotoxic effects of melphalan on marrow and gut crypt cells, but to a lesser extent, suggesting an enhanced therapeutic ratio.

The work described in this paper investigates further the chemosensitizing effect of MISO, using the two assay methods of cell survival and regrowth delay. We have worked principally with CY, but have also used melphalan. We have also used other 2-nitroimidazoles and compared their effectiveness with MISO, and have studied the influence of timing and drug dose on

* To whom reprint requests should be addressed.

chemosensitization. We report on the toxicity which we observed using several different endpoints.

### MATERIALS AND METHODS

*Tumours.*—The tumour used, denoted WHFIB, was a poorly differentiated sarcoma derived from a fibrosarcoma which arose spontaneously in inbred WHT mice. It was adapted for growth in culture (George *et al.*, 1977) so that *in vivo* treatments can be assayed either by cell survival *in vitro* or by tumour-growth delay.

*Methods of obtaining tumours.*—Tumours were grown either on the chest or as small lung tumours. S.c. tumours were obtained by first injecting $1\text{–}3 \times 10^7$ cultured cells into a mouse, and transplanting the resulting tumour 7–9 days later into the required number of mice, as described by George *et al.* (1977). Tumours reached treatment size, 6–8mm diameter, 12–18 days later. Small lung tumours were obtained by injecting $10^5$ viable cells together with $10^5$ heavily irradiated nonviable cells in a volume of 0·5 ml medium into the tail vein of a mouse. Nine days after injection, lung tumours were invisible to the naked eye, but 14 days after injection, 30–60 tumours of diameter up to 2 mm were visible on the pleural surface. If left intact the mouse would remain well for 15–18 days after injection, then become ill and have to be killed at 19–21 days.

*Cell-survival and growth delay assays.*—To assay cell survival, single-cell suspensions from s.c tumours or whole lungs were obtained as previously described (George *et al.*, 1977). The concentration of intact cells was then measured with a haemocytometer and a phase-contrast microscope. The appropriate number of cells was plated on to 50mm Petri dishes containing $5 \times 10^4$ heavily irradiated "feeder" cells, and the number of viable colonies was counted 9–10 days later. The plating efficiency was generally 25–40% for s.c. tumours and 20–35% for lung tumours.

For the growth-delay assay, tumours treated at 6–8mm mean diameter were measured 3 times weekly in 3 mutually perpendicular dimensions and the time calculated for each tumour to regrow to a geometric mean diameter either 2mm or 3·5mm larger than the treatment size. At treatment size the WHFIB tumour has a doubling time of about 3·5 days. The drugs were administered i.p. as a solution in normal saline. CY and melphalan were used within 10 min of dissolving them. Melphalan was first dissolved in a few tenths of a ml of 2% HCl in ethanol before being diluted in normal saline to the desired concentration. For each experiment the chemotherapeutic drugs were diluted such that a 30g mouse would receive either 0·3 or 0·6 ml. The radiosensitizing agents MISO, Ro 03-8799, Ro 12-5272 and Ro 05-9963 were dissolved at a concentration such that a 30g mouse would receive 1 ml.

### RESULTS

### *Subcutaneous tumours*

Fig 1 shows the survival of cells from s.c. WHFIB tumours after various doses of CY. Survival depended on the time between treatment and the excision of the tumour. Fig 1A shows that, in spite of the scatter, at each dose tumours excised at 2 h (open symbols) had a lower cell survival than tumours left *in situ* for 22–24 h (closed symbols). Taking mean values we see that after a dose of 150 mg/kg CY, for example, cell survival rose by a decade (Fig. 1B). A rise of this magnitude cannot be due to repopulation, since the cell-cycle time is $\sim$12 h. We believe the increase represents repair of potentially lethal damage (PLD) in this system, as reported by Twentyman (1977) for the EMT6 tumour.

When tumours were excised at different times after a fixed dose (75 mg/kg) of CY the same phenomenon was seen (Fig. 2A); cell survival fell to a minimum at 2–4 h and then increased. Other experiments (data not shown) have shown that most of the rise in survival was completed by 12 h. However, when 1 g/kg MISO was given and then 75 mg/kg CY 1 h later, this rise in survival was not seen (Fig. 2A). MISO potentiated cell kill by CY at 2–4 h but caused greater potentiation at 20 h, due to this apparent inhibition of PLD recovery. At 20 h the cell survival in tumours treated by combined MISO + CY was 50–100 times less than that in tumours treated by CY alone. MISO alone

(A) (B)

FIG. 1.—Cell survival in WHFIB tumours treated with CY: ○, at 2 h; ●, at 24 h. A, all values; B, mean values.

FIG. 2.—Cell survival in WHFIB tumours after: A: ●, 75 mg/kg CY only; ○, 1 mg/g MISO—1 h—75 mg/kg CY; △, 1 mg/g MISO only. B, 24 h after; ○, CY only, varying doses; △, 1 mg/g MISO —1 h—CY.

FIG. 3.—Time for WHFIB tumours to increase in mean diameter by 2 mm after: no treatment; 1 mg/g MISO only; 100 mg/kg CY only; or 1 mg/g MISO—1 h—100 mg/kg CY.

caused slight cell killing at 4 h in this experiment (Fig. 2A), but this was not a general finding and little cell kill was seen at later times.

When tumours were excised at 24 h after varying doses of CY preceded by 1 g/kg MISO, increased cell kill with the combined treatment was again seen (Fig. 2B). The lines through the data points of Fig. 2B were fitted by regression analysis, assuming exponential survival curves. With this assumption, MISO was dose-modifying with a dose-modifying factor of $2{\cdot}0 \pm 0{\cdot}4$ (95% confidence limits).

The regrowth-delay assay was also used to assess the potentiating effect of MISO on CY cytotoxicity. Tumours were treated with 100 mg/kg CY with or without 1 g/kg MISO given 1 h earlier. The results are shown in Fig. 3. CY alone caused 2·9 days delay in regrowth, whilst MISO alone caused none. However, MISO and CY together caused 12·6 days delay in regrowth, *i.e.* the MISO had "chemosensitized" the tumours to the action of CY to a highly significant extent.

We investigated the effects of other radiosensitizers which differed from MISO in their radiosensitizing efficiency and lipophilicity. These were, in order of decreasing radiosensitizing efficiency *in vitro*, Ro 03-8799, Ro 12-5272, MISO and Ro 05-9963 (Smithen *et al.*, 1980; Adams *et al.*, 1979) (Table). The interval between giving the radiosensitizers and CY was in each case taken as the time at which optimal radiosensitization in mouse tumours would be observed. For MISO this was 1 h, for Ro 03-8799 40 min and for Ro 05-9963 and Ro 12-5272 30 min (McNally *et al.*, 1978*b*; McNally, unpublished). The dose of CY was always 75 mg/kg for survival assays and 100 mg/kg for growth-delay assays. Fig. 4A shows that 1 g/kg Ro 03-8799 potentiated CY toxicity when tumours were excised at 4 h, and there was a further fall in survival if excision was delayed for 22 h, so that survival was then 2 decades lower in tumours receiving CY only. Ro 05-9963 at 1·5 g/kg increased the cell kill by CY by over a decade at 4 h, but some rise in survival was seen by 24 h (Fig. 4B). Neither of these 2-nitroimidazoles caused significant cell killing when used alone.

Fig. 5 shows the effects of the different sensitizers combined with CY on growth delay. The effect of the combination of sensitizer plus CY can be expressed in term of the "excess delay", defined as the growth delay due to the combination minus that due to CY alone. The excess delay for CY ranged from 12·3 days with 1 g/kg

TABLE

| Sensitizing agent | Mol wt | $E_7^1$/mV | Partition coefficient | Acute $LD_{50}$(g/kg) | Dose (g/kg) | Excess delay (days) | Relative chemosens. efficiency* |
|---|---|---|---|---|---|---|---|
| Misonidazole | 201·2 | −389 | 0·43 | 2·0 | 1·0 | 12·3 | 1 |
| Ro 03-8799 | 290·8 | −346 | 8·5 | 1·8 | 1·0 | 10·1 | 1·2 |
| Ro 12-5272 | 219·2 | −368 | 0·05 | — | 0·2 | 5·6 | 2·5 |
| Ro 05-9963 | 187·2 | −389 | 0·11 | 3·8 | 1·0 | 2·2 | 0·2 |

* See text.

FIG. 4.—Cell survival in WHFIB tumours after: A: ●, 75 mg/kg CY only; ○, 1 mg/g Ro 03-8799—40 min—75 mg/kg CY; △, 1 mg/g Ro 03-8799 only. B: ●, 75 mg/kg CY only; ○, 1·5 mg/g Ro 05-9963—30 min —75 mg/kg CY; △, 1·5 mg/g Ro 05-9963 only.

FIG. 5.—Time for WHFIB tumours to increase in mean diameter by 2 mm after treatments as shown. Dose of CY, 100 mg/kg in each case.

FIG. 6.—Excess delay, *i.e.* the time to increase in mean diameter by 2 mm after combined treatment minus the time to increase in mean diameter by 2 mm after CY alone, in WHFIB tumours following: A, varying doses of MISO 30 min before 100 mg/kg CY; B, varying doses of Ro 05-9963 30 min before 100 mg/kg CY.

MISO to 2·2 days with 1 g/kg Ro 05–9963. These values cannot be compared directly, because the tumour concentration of the sensitizers will be different and they have different toxicities. Ro 12–5272 was relatively insoluble, and this limited its dose to 0·2 g/kg. An approximately linear relationship between excess delay and dose of radiosensitizer was obtained for

FIG. 7.—Excess delay to increase in mean diameter by 2 mm following: 0·8 mg/g MISO at varying intervals before or after 100 mg/kg CY.

MISO and, with less certainty, for Ro 05-9963 (Fig. 6).

In order to determine the effect of the interval between MISO and CY, MISO was given to tumour-bearing mice either 6 h or 1 h before CY, or simultaneously, or 2 h after CY. The results are shown in Fig. 7, in which the excess delay is plotted against the interval between the drugs. The maximum effect was seen when the MISO was injected 1 h before CY, as might have been expected if the effect is largest when CY is given at maximum MISO concentration in the tumour. There was still a considerable effect when it was given 6 h before, and a small, but significant effect when it was given 2 h after the CY. In this experiment the MISO dose was reduced to 0·8 g/kg.

Fig. 8 shows the effect of CY alone or in combination with MISO on tumours up to 2 mm diameter growing in mouse lungs. The animals were treated 16–18 days after injection of the cells, and the lungs were excised at various times after injection of CY. Fig. 8A shows that, after a dose of 75 mg/kg, CY survival was the same whether lungs were excised at 4–6 or 20 h, in-

(A) (B)

FIG. 8.—Cell survival in WHFIB lung tumours after: A: ●, 75 mg/kg CY; ○, 1 mg/g MISO—1h—75 mg/kg CY; △, 1 mg/gMISO only. B: Cell survival in WHFIB lung tumours excised 4–6 h after: ○, CY only; △, 1 mg/g MISO—1 h—CY. DMF = 1·4.

FIG. 9.—Cell survival in WHFIB tumours after: A: ●, 5 mg/kg melphalan only; ○, 1 mg/g MISO—1 h—5 mg/kg melphalan; △, 1 mg/g MISO only. B: 24 h after: ○, melphalan only, varying doses; 1 mg/g MISO—1 h—melphalan, varying doses. DMF = 2·7.

FIG 10.—White cell counts in 1000s/per mm$^3$ after treatment with: A: ○, 100 mg/kg CY only; ●, 1 mg/g MISO—1 h—100 mg/kg CY. B: ◇, 150 mg/kg CY only; ◆ 1 mg/g MISO—1 h—150 mg/kg CY. C: △, 225 mg/kg CY only, ▲, 1 mg/g MISO—1h—225 mg/kg CY. D: □, no treatment; ■, 1 mg/g MISO only.

dicating no recovery from PLD. In combination with 1 g/kg MISO given 1 h before CY, there was an enhancement of cell kill at 4 and 6 h, with probably no further change at 20 h. The single point at 20 h was because the combination of the 2 drugs was found to be quite toxic to mice bearing 16–18-day-old lung colonies, in contrast to those bearing s.c. tumours. This meant that 2/4 mice receiving both MISO and CY died within 20 h. (In one of those that survived, the tumour-cell yield was unexpectedly low.) Thus the data in Fig. 8 are limited. Nevertheless, it implies that there was probably no recovery from potentially lethal drug damage in these small lung tumours. Because of this, and the toxicity of the combined treatment, full dose–effect curves were determined for tumours excised 4–6 h after treatment (Fig. 8B). They have been corrected for the toxicity of MISO alone, which reduced the surviving fraction to 0·53 at 4–6 h. Comparison with Fig. 2 shows that the cells were more sensitive in the lung tumours than in s.c. tumours. The lines have been fitted assuming exponential inactivation and give a dose-modifying factor (with 95% confidence limits) of $1{\cdot}40 \pm 0{\cdot}38$.

The effect of MISO on the cytotoxicity of melphalan was also studied. Fig. 9A shows the survival of cells from WHFIB tumours as a function of excision time after a dose of 5 mg/kg melphalan, either alone or preceded by 1 g/kg of MISO. After melphalan alone (closed circles) there was a small rise in cell survival between 4 and 22 h. When 1 g/kg MISO was injected 1 h before melphalan (open circles) there was potentiation of the cell kill at 4 h, and a greater potentiation at 22 h. Circles with downward arrows refer to points at which no colonies were counted, and have been calculated as for one colony. Fig. 8B shows survival curves for cells from tumours excised at 24 h after varying doses of melphalan, either alone or preceded by 1 g/kg MISO. The lines have been fitted by regression analysis, assuming exponential inactivation. MISO was dose-modifying with a DMF of $2{\cdot}7 \pm 0{\cdot}7$ (95% confidence limits).

*Normal tissue toxicity*

In non-tumour-bearing mice, 1 g/kg of MISO 1 h before CY caused no significant lowering of the $LD_{50/60}$ of 260 mg/kg due to CY alone.

Cystitis is a well-known side effect of CY treatment, and is caused by damage to the bladder epithelium from contact with acrolein (Cox, 1979). We therefore attempted to assess whether cystitis was increased by MISO treatment. The system of Stewart *et al.* (1978) was used to measure the hourly urination frequency of male WHT mice. Mice were treated with 75 or 125 mg/kg CY, either alone or preceded by 1 g/kg MISO 1 h earlier, with appropriate controls. The urination frequency of groups of 5 mice was measured continuously for 10 days, then weekly up to one month. 75 mg/kg CY caused a marked increase in frequency, which lasted for 10 days. MISO decreased this frequency. In mice receiving 125 mg/kg CY, there was no increase in frequency over controls (perhaps because of their reduced fluid intake), yet their frequency was *still* decreased by MISO. Mice treated with MISO alone had a lower frequency then untreated controls. After 2 weeks the frequencies of all groups were the same. These findings suggest that while MISO did not sensitize the bladder epithelium to CY damage, at least in terms of an increasing urination frequency, there may have been effects on fluid uptake. These would complicate the interpretation of this result at the higher drug dose, and so we cannot state unequivocally that there was no effect of MISO on CY toxicity to the bladder, though it appears to be minimal.

Leucopenia (fall in white-cell count) and thrombocytopenia (fall in platelet count) are important haematological side-effects of CY. Samples of mouse tail blood were taken to assess whether MISO increased the leucopenia associated with a dose of CY. After a dose of CY the white-cell count fell to a nadir at 4 days, and returned to normal levels by 2–4 weeks (Fig. 10A, B, C). If CY injection was preceded by 1 g/kg of MISO 1 h earlier, there was no further effect on the white-cell count; MISO alone had no effect (Fig. 10D). Thus, at therapeutic levels, there was no enhancement by MISO of the effect of CY on white-cell count.

### DISCUSSION

Fig. 1 shows that in the s.c. WHFIB tumour there was a rise in cell survival between 2 and 24 h after a dose of CY. This rise was too large to be accounted for by repopulation. We believe that it is due to repair of PLD, as reported earlier by Twentyman (1977) using the EMT6 tumour. It follows from this that measurements of tumour cell survival 1 day after treatment are more likely to be correlated with *in situ* tumour response than those measured within a few hours, and so are probably more relevant to the clinical situation.

The 4 radiosensitizing drugs tested are all of similar electron affinity (Table). Radiobiological data on V79 cells *in vitro* have shown MISO and Ro 05-9963 to be of equal radiosensitizing efficiency, but Ro 03-8799 and Ro 12-5272 are more effective *in vitro* (Flockhart *et al.*, 1978*b*; Adams *et al.*, 1979; Smithen *et al.*, 1980; Watts *et al.*, 1980).

Ro 03-8799 sensitized tumours to Cy at 4 and 22 h after treatment (Fig. 4A), probably to the same extent as MISO (Fig. 2A). In these particular experiments the CY controls did not clearly show the rise in survival with time seen in other experiments, but the survival in tumours receiving combined treatment nevertheless fell between 4 and 22 h, so that chemosensitization was much greater at 22 h than at 4 h. For Ro 05-9963, Fig. 4B shows that cell survival rose between 4 and 22 h after combined treatment. This, combined with the fact that the molar dose was greater for Ro 05-9963 than for Ro 03-8799, suggests that Ro 05–9963 is a less effective chemosensitizer (see below).

The tumour-growth delays were in accordance with the *in vitro* data (Fig. 5). The dose of Ro 12-5272 was only 0·2 g/kg, because of its poor solubility. Ro 03-8799 has a mol.wt of 290·8, compared with 201·2 for MISO (Table) so a dose of 1 g/kg for Ro 03-8799 represents in molar terms only 69% of a dose of 1 g/kg of MISO. With MISO and Ro 05-9963 the dose–response curves were nearly linear (Fig. 6). If we assume that this is so for all 4 radiosensitizing drugs, we can correct for dose and molarity to give a relative "chemosensitizing efficiency" compared with MISO, for a chemosensitizing drug Y, thus:

relative "chemosensitizing efficiency of drug Y is:

$$\frac{\text{excess delay with Y}}{\text{excess delay with MISO}} \times \frac{\text{dose of MISO}}{\text{dose of Y}} \times \frac{\text{mol. wt Y}}{\text{mol. wt MISO}}$$

The results of such calculations are shown in the Table. On this basis Ro 03-8799 was a marginally better chemosensitizer than MISO. Ro 12-5272 was clearly more effective than MISO. Its mol.-wt is similar to that of MISO (Table) so a direct comparison can be made between the two drugs. Figs. 5 and 6 show that whereas 0·2 mg/g MISO would have caused virtually no extra growth delay the same dose of Ro 12-5272 caused 5 days extra delay. Ro 05-9963 was the least effective sensitizer.

When the interval between the injection of MISO and of CY was varied the extra growth delay was best when MISO was injected 1 h before CY (Fig. 7). However, significant chemosensitization was also found when MISO was given 6 h before CY or even 2 h after. The tumour half-life of MISO is short (McNally *et al.*, 1978b), so after 6 h a very small amount of the MISO would be left in the tumour. It follows that the large effect seen when MISO is given 6 h before CY must be a consequence of the damage done to the cell by the MISO, and not of the quantity of MISO left in the cell at 6 h. Similarly, the CY-induced damage must still be capable of interaction with MISO 2 h later.

MISO also sensitized the action of melphalan on s.c. WHFIB tumours (Fig. 9). The cell kill was potentiated at both 4 h and 22 h after treatment. There was a rise in survival between 4 and 22 h in tumours treated with melphalan alone, again suggesting PLD repair (Fig. 9B). Because of the excessive cell kill it is not clear whether or not PLD repair was inhibited by MISO. Stratford *et al.* (1980) have found that MISO potentiates the action of *cis*-platinum, which also acts as an alkylating agent. Thus it is possible that MISO and other 3-nitroimidazoles sensitize tumours *in vivo* to the action of the whole group of alkylating agents.

Small WHFIB lung tumours did not show repair of PLD after CY (Fig. 8), and there was less chemosensitization by MISO than in the s.c. tumours. The treatment was more toxic to mice bearing lung tumours than to mice bearing s.c. tumours of comparable cell numbers. Five out of 11 mice treated with doses of 25, 50 or 75 mg/kg CY plus 1 mg/g MISO died within 20 h and 2/5 treated with MISO alone died. No mice bearing s.c. tumours showed any significant ill health as a result of treatment with MISO or CY or the two together. These experiments were performed 16–18 days after i.v. injection of tumour cells, at which time the mice generally appeared well, but contained $10^7$–$10^8$ tumour cells in their lungs. These would exert more adverse physiological effects than $10^7$–$10^8$ cells in an s.c. tumour. It is perhaps not surprising therefore, that the stress of the injections of MISO and CY was more toxic to them.

The lack of normal-tissue toxicity due to the combined treatment has been encouraging. The $LD_{50/60d}$ of CY in non-tumour-bearing mice was not lowered significantly by MISO. Frequency of urination was not increased. The white-cell counts after therapeutic levels of CY were not significantly altered by 1 g/kg MISO given 1 h earlier. It is possible that tumour-bearing mice might be more affected by the stress of the double treatment than non-tumour-bearing mice, owing to the altered physiology in malignant disease, so that the $LD_{50}$ and blood counts, for example, might show more difference between the combined treatment and CY-only in tumour-bearing animals than in our experiments in non-tumour-bearing animals. The toxicity in mice bearing lung tumours suggests that the combination should be used with caution in ill patients or those with lung metastases.

It must be borne in mind that the MISO doses used throughout these experiments were higher than those that can be used in the clinic. For instance, in the 6-fraction regime of Dishe *et al.* (1977) they used a total of 12 g/m$^2$, which is equivalent to about 0·05 g/kg fraction. Thus the degree of enhancement in human tumours may be less than that seen in these experiments. However, the half-life of MISO in the human is long compared with the mouse (Flockhart *et al.*, 1978*a*, *b*), which could be advantageous. Also, standard chemotherapy regimes often require drugs to be injected one or twice monthly, for instance so that it might be possible to use higher doses of MISO with these longer intervals. Alternatively, it may be possible to use Ro 03-8799, or to design other drugs which might be less toxic than MISO yet which would be equally effective as chemosensitizers.

The mechanisms whereby MISO enhanced the action of CY and melphalan are not known. It is unlikely that there were such significant alterations in the pharmacokinetics of these two drugs that overall exposure of the tumours increased sufficiently to cause such enhanced cell kill. Since MISO potentiated the action of CY more on tumour than on normal tissues, hypoxic conditions may be necessary for chemosensitization.

If chemosensitization were possible in clinical cancer, the opportunity might exist for improving the treatment of metastatic disease. We have shown that good sensitization is possible, but only at sensitizer doses beyond the clinical range used in radiotherapy. However, it may be possible to find a combination of chemosensitizer and cytotoxic agent which could be effective on tumours at doses within the clinical range.

We should like to thank the Medical Research Council for the Fellowship which enabled this work to be carried out and the Cancer Research Campaign for financial support.

We are grateful to Ward-Blenkinsop (Bracknell) for supplying cyclophosphamide, the Wellcome Foundation for supplying melphalan and to Roche Products Ltd (Welwyn) for supplying the radiosensitizing drugs. We wish to thank Mrs Lynda Hall and Mr Peter Russell and their colleagues for care of the animals. We are grateful to Professor J. Fowler for enthusiastic encouragement, and to Dr Peter Wardman and Mr Eric Clarke for the physicochemical details of the radiosensitizers. We are especially grateful to Miss Margeret Hinchliffe for expert technical assistance.

### REFERENCES

ADAMS, G. E., CLARKE, E. D., FLOCKHART, I. R. & 8 others (1979) Structure–activity relationships in the development of hypoxic cell radiosensitizers. I. Sensitization efficiency. *Int. J. Radiat. Biol.*, **35**, 133.

ADAMS, G. E., FLOCKHART, I. R., SMITHEN, C. E., STRATFORD, I. J., WARDMAN, P. & WATTS, M. E. (1976) Electron-affinic sensitization. VII. A correlation between structures, one-electron reduction potentials and efficiencies of nitroimidazoles as hypoxic cell radiosensitizers. *Radiat. Res.*, **67**, 9.

ANDERSON, R. F. & PATEL, K. B. (1979) Effect of lipophilicity of nitroimidazoles on radiosensitization of hypoxic bacterial cells *in vitro*. *Br. J. Cancer*, **39**, 705.

BROWN, J. M. (1977) Cytotoxic effects of the hypoxic cell radiosensitizer Ro 07-0582 to tumour cells *in vivo*. *Radiat. Res.*, **72**, 469.

COX, P. J. (1979) Cyclophosphamide cystitis: Identification of acrolein as the causative agent. *Biochem. Pharmacol.*, **28**, 2045.

DENEKAMP, J. (1978) Cytotoxicity of radiosensitization in mouse and man. *Br. J. Radiol.*, **51**, 636.

DENEKAMP, J. & McNALLY, N. J. (1978) The magnitude of hypoxic cell cytotoxicity of misonidazole in human tumours. *Br. J. Radiol.*, **51**, 747.

DISCHE, S., FOWLER, J. F., SAUNDERS, M. I., & 4 others (1980) A drug for improved radiosensitization in radiotherapy. *Br. J. Cancer*, **42**, 153.

DISCHE, S., SAUNDERS, M. I., LEE, M. E., ADAMS, G. E. & FLOCKHART, I. R. (1977) Clinical testing of the radiosensitizer Ro 07-0582: Experience with multiple doses. *Br. J. Cancer*, **35**, 567

FLOCKHART, I. R., LARGE, P., TROUP, D., MALCOLM, L. & MARTEN, T. R. (1978*a*) Pharmaco-kinetic and metabolic studies of the hypoxic cell radiosensitizer misonidazole. *Xenobiotica*, **8**, 97.

FLOCKHART, I. R., SHELDON, P. W., STRATFORD, I. J. & WATTS, M. E. (1978*b*)A metabolite of the 2-nitroimidazole misonidazole with radiosensitizing properties. *Int. J. Radiat. Biol.*, **34**, 91.

GEORGE, K. C., HIRST, D. G. & McNALLY, N. J. (1977) Effects of hyperthermia on cytoxicity of the radiosensitizer Ro 07-0582 in a solid mouse tumour. *Br. J. Cancer*, **35**, 372.

HALL, E. J. & ROIZIN-TOWLE, L. (1975) Hypoxic sensitizers: Radiobiological studies at the cellular level. *Radiology*, 117, 453.

McNALLY, N. J., DENEKAMP, J., SHELDON, P. W. & FLOCKHART, I. R. (1978*a*) Hypoxic cell sensitization by misonidazole *in vivo* and *in vitro*. *Br. J. Radiol.*, **51**, 317.

McNALLY, N. J., DENEKAMP, J., SHELDON, P. W., FLOCKHART, I. R. & STEWART, F. A. (1978*b*). Radiosensitization by misionidazole (Ro 07-0582): The importance of timing and of tumour concentrations. *Radiat. Res.*, **73**, 568

MARTIN, W. M. C. & McNALLY N. J. (1980) The cytotoxic action of adriamycin on tumour cells *in vitro* and *in vivo*. *Br. J. Cancer*, **41** (Suppl. IV), 306.

ROIZIN-TOWLE, L. & HALL, E. J. (1978) Studies with bleomycin and misonidazole on aerated and hypoxic cells. *Br. J. Cancer*, **37**, 254.

ROSE, C. M., MILLAR, J. L., PEACOCK, J. H., PHELPS, T. A. & STEPHENS, T. C. (1980) Differential enhancement of chemotherapy cytotoxicity in tumor and normal tissue by misonidazole. *Proc. Am. Assoc. Cancer Res.*, **21**, 264.

SMITHEN, C. E., CLARKE, E. D., DALE, J. A. & 4 others (1980) Novel (nitro-1-imidazolyl) alkanolamines as potential radiosensitizers with improved therapeutic properties. In *Radiation Sensitizers: Their Use in the Clinical Management of Cancer*. New York: Masson. p. 22

STEWART, F. A., MICHAEL, B. D. & DENEKAMP, J. (1978) Later radiation damage in the mouse bladder as measured by increased urination frequency. *Radiat. Res.*, **75**, 649.

STRATFORD, I. J., ADAMS, G. E., HORSEMAN, M. R. & 4 others (1980) The interaction of misonidazole with radiation, chemotherapeutic agents or heat. *Cancer Clinical Trials* **3**, 231.

SUTHERLAND, R. M. (1974) Selective chemotherapy of non-cycling cells in an *in vitro* tumour model. *Cancer Res.*, **34**, 3501.

TWENTYMAN, P. R. (1977) Sensitivity to cytotoxic agents of the EMT6 tumour *in vivo*: Tumour volume *versus in vitro* plating. 1. Cyclophosphamide. *Br. J. Cancer*, **35**, 208.

URTASUN, R. C., BAND, P. R., CHAPMAN, J. D., RABIN, H., WILSON, A. F. & FRYER, C. G. (1977) Clinical Phase I study of the hypoxic cell radiosensitizer Ro 07-0582 2-nitroimidazole derivative. *Radiology*, **122**, 801.

WATTS, M. E., ANDERSON, R. F., JACOBS, R. S. & 7 others (1980) Evalution of novel hypoxic cell radiosensitizers *in vitro*: The value of studies in single-cell systems. In *Radiation Sensitizers: Their Use in the Clinical Management of Cancer*. New York: Masson. p. 175.